# The clinical efficacy of fluticasone propionate combined with ACEI/ARB in the treatment of immunoglobulin A nephropathy

**DOI:** 10.1186/s12882-023-03106-4

**Published:** 2023-03-22

**Authors:** Liping Sun, Xinyi Zi, Zhen Wang, Xinzhou Zhang

**Affiliations:** 1 Department of Nephrology, Shenzhen Key Laboratory of Renal, Shenzhen People’s Hospital, The Second Clinical Medical College, Jinan University, Guangzhou, China; 2 The First Affiliated Hospital, Southern University of Science and Technology, Shenzhen, Guangdong 518020 China

**Keywords:** IgA nephropathy, Fluticasone propionate aerosol, Proteinuria, Clinical efficacy

## Abstract

**Background:**

Immunoglobulin A nephropathy (IgAN) is the most common primary glomerulopathy worldwide, and lacks the effective treatment. The study was aimed to investigate the clinical efficacy of fluticasone propionate aerosol combined with angiotensin converting enzyme inhibitor / angiotensin receptor blocker (ACEI/ARB) in the treatment of IgAN.

**Methods:**

142 patients with biopsy-proven IgAN at Shenzhen People?s hospital from June 2018 to June 2020 were enrolled. The patients were randomly divided into the supportive care plus fluticasone group and the supportive care group. The patients of the supportive care plus fluticasone group were treated with fluticasone propionate aerosol (250 ?g Bid) combined with ACEI/ARB, while the supportive care group was merely treated with ACEI/ARB. The patients were followed up at 3, 6 and 9 months after enrollment. Primary outcomes include changes in proteinuria and estimated glomerular filtration rate (eGFR).

**Results:**

The level of proteinuria in the supportive care plus fluticasone group was significantly lower compared with the supportive care group at 0, 3, 6 and 9 months. Meanwhile, during the follow-up period, no serious adverse events were recorded during the study in either group. However, fluticasone treatment did not alleviate the decline in eGFR.

**Conclusion:**

Fluticasone propionate aerosol combined with ACEI/ARB can reduce the level of proteinuria in thetreatment of IgAN, and has no significant effects on renal function.

## Introduction

Immunoglobulin A nephropathy (IgAN) was first described by Jean Berger in 1968 as a kind of primary glomerulopathy, in which immunoglobulin A (IgA) predominantly deposits in glomerular mesangium. Subsequent researches have manifested that IgAN is the most common primary glomerulopathy worldwide, and it is one of the causes of chronic kidney disease (CKD) and end-stage kidney disease (ESRD) [1, 2]. The incidence of IgAN in China is approximately 24% and about 20%~40% of patients will develop into ESRD within 10 ~ 20 years [3]. However, the efficacy of corticosteroids is controversial, and adverse side effects are well documented [4, 5]. At present, the use of rituximab in IgAN therapy has limited evidence, and has not achieved satisfactory efficacy in clinical randomized trials [6]. Angiotensin-converting enzyme inhibitors (ACEI) or angiotensin receptor blockers (ARBs) are antihypertensive drugs and the most commonly non-immunosuppressive drugs [7]. In patients with IgAN, ACEI and ARBs could reduce the passage of macromolecules through the glomerulus [8], thereby reducing the level of proteinuria, but with the side effect of dizziness [9]. Therefore, some novel, effective and safe therapeutic options need to be addressed.

It has been observed that IgAN is mediated by mucosal immunization since the 1970s. Upper respiratory tract infections could aggravate clinical manifestations of IgAN in the early stage. The episodes of macroscopic hematuria associated with upper respiratory tract infections are common in these patients. It has been reported that various pathogens in the upper respiratory tract activate the immunological system by giving rise to tonsillitis, which is involved in the pathogenesis of IgAN [4]. Fluticasone propionate, similar to glucocorticoid, has the function of anti-inflammation. It could inhibit inflammatory lymphocytes and inflammatory mediators they release, so that hinder inflammatory cells on the surface of the airway from being generated or activated [10].

The present study was designed to provide an estimation of the efficacy and safety assessment of fluticasone propionate aerosol in IgAN and help in determining whether a larger multicenter trial with clinical outcomes is warranted.

## Materials and methods

### General information

**Fig. 1:**
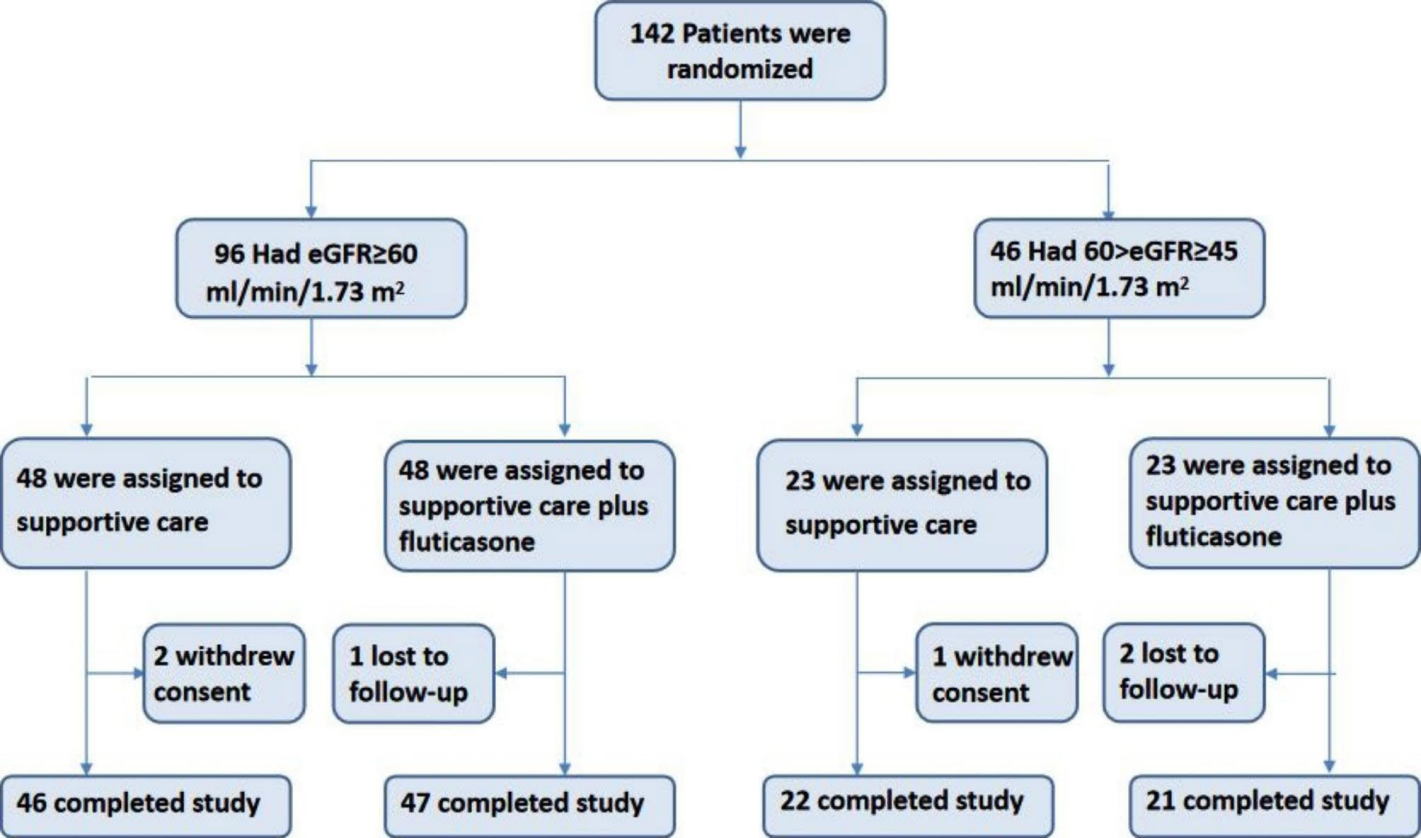
The chart of experimental flow A total of 142 patients who had completed the three-month break-in phase participated in the subsequent 9-month trial phase. Of these, 96 had a eGFR of at least 60 ml/min/1.73m^2^, and 46 had a eGFR of 45 to 59 ml/min/1.73m^2^. These patients were randomly assigned to continue support therapy or receive additional fluticasone

142 patients with biopsy-proven IgAN at Shenzhen people’s hospital from June 2018 to June 2020 were enrolled. Inclusive criteria: (1) Age range is between 18 and 55; (2) Blood pressure is kept below 130/90 mmHg after 3-month treatment of maximum tolerable dose of ACEI/ARB; (3) estimated glomerular filtration rate (eGFR) calculated by using CKD-EPI formula is no less than 45ml/mim/1.73m^2^; (4) The quantity of the urinary protein ≥ 1.0 g/d or first-morning urine albumin-creatinine ratio (UACR) ≥ 0.8 g/g twice in a row. Exclusive criteria: (1) Secondary IgAN; (2) Using corticosteroid or immunosuppressive agents within the year before treatment; (3) Having a history of allergies to ACEI and or ARB; (4) Albumin < 30 g/L; (5) BP < 90/60 mmHg; (6) Plasma K^+^ > 5.5 mmol/L; (7) Acute respiratory infections; (8) Unilateral or bilateral renal artery stenosis; (9) Women who are pregnant or nursing; (10) Serious heart, brain, liver or hematopoietic system disorders or other nasty diseases influencing the survival; (11) Engaged in other clinical research. The experimental flow chart is shown in Fig. 1. Ethical approval to conduct this trial was obtained from the ethics committees of Shenzhen People’s Hospital (LL-KY-2,019,293). All subjects signed informed consent forms.

### Treatment method

This study was a prospective, double-blind, randomized, controlled study. In order to eliminate inter-group eGFR interference, we divided all patients into 2 modules according to the eGFR value (eGFR ≥ 60 ml/mim/1.73m^2^, 96 cases; and 45 ≤ eGFR < 60 ml/mim/1.73m^2^, 46 cases). Then, the patients in the two modules were randomly divided into two groups as followed: supportive care plus fluticasone group and the supportive care group (n = 71 in each group). The detailed procedures for grouping were displayed in Fig. 1. The patients in the supportive care plus fluticasone group were given fluticasone propionate inhalation aerosol (250 µg Bid) manufactured by GlaxoWellcome for the Basic treatment of ACEI/ARB. The supportive care group continued the original treatment option. Calcium Channel Blocks (felodipine sustained release tablets or nifedipine controlled release tablets) would be given to patients whose blood pressure is over 140/90 mmHg. Hyperlipidemia, infection and hypercoagulable states should be dealt with appropriately if necessary. Glucocorticoid and immunodepressants were prohibited in this study.

### Follow-up

The patients were followed up for 9 months after treatment. Three patients of the supportive care group withdrew from the study early due to withdrawal of informed consent. Three patients of supportive care plus fluticasone group were lost to follow-up (Fig. 1). The level of proteinuria was detected at 3, 6 and 9 months of follow-up, and the renal function was evaluated by eGFR. In addition, the adverse reactions and serious adverse events that occurred in the patients during the follow-up period were also recorded. Adverse events and severe adverse events are defined in accordance with Good Clinical Practice (GCP) issued by the Chinese food and drug administration (CFDA).

### Statistical methods

Statistical analysis was performed by SPSS 24.0 system software (SPSS, USA). All analyses were conducted according to the intention-to-treat principle. Count data were expressed as constituent ratio and the chi-square test was used for comparison. Continuous variables were expressed as the mean ± standard deviation (SD), non-normally distributed data were presented as median with interquartile range (IQR). The t-test was used for the comparison of normally distributed continuous variables, while the Mann-Whitney U test was applied for the comparison of non-normally distributed continuous variables. An independent samples t-test was used on variables of the two groups compared. *P* < 0.05 was considered statistically significant.

## Results

### Baseline characteristics

From June 2018 to June 2020, a total of 142 potentially eligible patients were screened for the study and underwent random assignment. Among 142 patients, 96 cases (68%) had eGFR ≧ 60ml/min/1.73 m^2^, and the eGFR of 46 cases (32%) was 45–59 ml/min/1.73m^2^ (Fig. 1). 71 cases were assigned to supportive care and 71 cases were assigned to supportive care plus fluticasone propionate inhalation aerosol. Overall, the comparison of baseline characteristics between groups showed no statistically significant difference (Table 1).

**Table 1:** Baseline characteristics of patients

Characteristics	Supportive Care (n = 71)	Supportive care plus fluticasone (n = 71)	*P*
Age (y)	39.3 ± 10.1	39.6 ± 10.5	0.9
BMI (kg/m^2^)	23.46 ± 4.16	23.96 ± 3.42	0.4
Female, n(%)	14 (19%)	18 (25%)	0.4
Smoking, n(%)	16 (22%)	14 (19%)	0.7
Systolic pressure	127 ± 8.4	125 ± 8.7	0.2
Serum creatinine (µmol/L)	120.2 ± 30.6	127.5 ± 35.7	0.2
eGFR (ml/min/1.73m^2^)	86.42 ± 33.93	84.2 ± 32.2	0.7
Daily urinary protein (g/d)	1.9 ± 0.4	1.8 ± 0.7	0.3
UACR (g/g)	0.88 ± 0.6	0.90 ± 0.5	0.5
Oxford histologic score			
M0/M1	9/17	12/16	0.5
E0/E1	16/10	20/8	0.4
S0/S1	3/23	12/16	0.01
T0/T1	13/8	14/12	0.6
Therapy with ACEI	25	22	0.6
Therapy with ARB	46	49	0.6

BMI, body mass index; eGFR, estimated glomerular filtration rate; UACR, urinary albumin-to-creatinine ratio; ACEI, angiotensin-converting enzyme inhibitor; ARB, angiotensin receptor blocker

### Impact of fluticasone propionate aerosol on the proteinuria of IgAN patients

The curative effect was evaluated by detecting the level of proteinuria in the two groups after treatment. The results showed that the level of proteinuria in the patients gradually decreased during 9 months after fluticasone propionate aerosol combined with Supportive care treatment, and was significantly lower than that of the Supportive care group (Table 2). It indicates that fluticasone propionate aerosol may have a good therapeutic effect on patients with IgAN.

**Table 2:** Comparison of proteinuria between two groups at different time points

Follow-up time (months)	Supportive care group (n = 71, g/d)	Supportive care plus fluticasone group (n = 71, g/d)	*p*
3	1.9 [1.5, 2.5]	1.2 [0.8, 1.7]	0.001
6	2.0 [1.1, 2.5]	1.0 [0.7, 1.4]	< 0.001
9	1.8 [0.9, 2.6]	0.9 [0.5, 1.0]	0.002

### Impact of fluticasone propionate aerosol on the renal function of IgAN patients

To further evaluate the effect of fluticasone propionate aerosol on renal function by detecting the eGFR of the two groups during the follow-up period. The results showed that there was no significant difference in the eGFR decline between the two groups at 3, 6 and 9 months after treatment (Table 3). In addition, the patient rinsed with water after each inhalation of fluticasone propionate, and no obvious local or systemic adverse reactions were observed (i.e., any increase in liver enzymes or differences in blood cell counts, including leukopenia, and immunosuppressive combined malignant tumors).

**Table 3:** Comparison of eGFR decline from baseline in the two groups at different time points

Follow-up time (months)	Supportive care group (n = 71)	Supportive care plus fluticasone group (n = 71)	*p*
3	1.5 [-4.4, 11.4]	-1.5[-12.3, 3.4]	0.1
6	2.5 [-7.0, 9.4]	-3.2 [-11.6, 10.9]	0.3
9	0.0 [-12.6, 19.3]	4.5 [-12.3, 23.1]	0.9

## Discussion

In this trial, we found that fluticasone propionate combined with ACEI/ARB effectively decreased urinary protein level, but did not delay eGFR decrease in IgAN patients with biopsy-proven with mild to moderate proteinuria.

It is known that macroscopic hematuria and/or increase of urinary protein occur in most IgAN patients after upper respiratory tract or alimentary tract infection, which implies that the development and progress of IgAN is closely related to immune system dysfunction [11–13]. Previous studies have reported that the level of hematuria and/or proteinuria improved after tonsillectomy in IgAN patients with recurrent tonsillitis [14]. Furthermore, research has discovered that RSV infection could result in more severe pathological changes in the kidneys of IgAN mice with a significant increase of proinflammatory Th cells and its inflammatory factors released [15]. Therefore, regulating the mucosal immune function of the upper respiratory tract may play a protective role in renal prognosis of IgAN patients. This is consistent with the results of this study.

Fluticasone propionate has the function of anti-inflammation to inhibit inflammatory lymphocytes and inflammatory mediators they release, so that it could hinder inflammatory cells on the surface of the airway from being generated or activated [10]. In in vitro studies, the affinity of fluticasone propionate to human glucocorticoid receptor is 18 times higher than that of dexamethasone, and over 3 times higher than budesonide [16, 17]. In our study, fluticasone propionate aerosol could reduce the level of urinary protein in IgAN patients. Growing evidence suggests that the descending rate of eGFR will slow down if the urinary protein decreases even slightly [4, 18, 19]. But no reduction in eGFR was observed in our study after combination therapy, suggesting that the treatment option of fluticasone propionate combined with ACEI/ARB is not good for slowing the decline of eGFR in IgAN patients.

Compared with other similar glucocorticoids, fluticasone propionate is less absorbed from the gastrointestinal tract and has a significant liver first pass effect. After inhalation, the dose of fluticasone propionate into the circulatory system through swallowing is almost zero [20]. In our study, no adverse events such as impaired glucose intolerance, steroid diabetes, blood pressure rising, weight gain and severe infections were observed in patients after fluticasone propionate inhalation, indicating that the drug has good safety and tolerability in IgAN patients.

There are several limitations of this study. The primary limitation was the size and duration of the trial. It was a small-sample and single-center trial with short follow-up. Additionally, no hard endpoint was met. A larger trial of longer duration may demonstrate differences in endpoint. Therefore, further studies are needed to see if fluticasone propionate aerosol is useful for improving long-term outcomes for IgAN patients. This study demonstrated that fluticasone propionate combined with ACEI/ARB can effectively lower urinary protein level in IgAN patients even if the eGFR decrease is not delayed, and no adverse events were observed, which suggests that fluticasone propionate may provide a new treatment option for IgAN patients.

## Conclusion

In conclusion, this study found that inhalation of fluticasone propionate could significantly reduce the level of proteinuria in IgAN patients, but not eGFR. This suggests that fluticasone propionate can treat IgAN to a certain extent and should be further investigated.

## Data Availability

Datasets used in this article are available from corresponding author on reasonable request.
